# Embryonic lethality is not sufficient to explain hourglass-like conservation of vertebrate embryos

**DOI:** 10.1186/s13227-018-0095-0

**Published:** 2018-03-16

**Authors:** Yui Uchida, Masahiro Uesaka, Takayoshi Yamamoto, Hiroyuki Takeda, Naoki Irie

**Affiliations:** 1 Department of Biological Sciences, University of Tokyo, 7-3-1 Hongo, Bunkyo-ku, Tokyo, 113-0033 Japan; 2 Universal Biology Institute, University of Tokyo, 7-3-1 Hongo, Bunkyo-ku, Tokyo, 113-0033 Japan

**Keywords:** Developmental conservation, Hourglass model, Phylotypic period, Lethality, Pharyngula period

## Abstract

**Background:**

Understanding the general trends in developmental changes during animal evolution, which are often associated with morphological diversification, has long been a central issue in evolutionary developmental biology. Recent comparative transcriptomic studies revealed that gene expression profiles of mid-embryonic period tend to be more evolutionarily conserved than those in earlier or later periods. While the hourglass-like divergence of developmental processes has been demonstrated in a variety of animal groups such as vertebrates, arthropods, and nematodes, the exact mechanism leading to this mid-embryonic conservation remains to be clarified. One possibility is that the mid-embryonic period (pharyngula period in vertebrates) is highly prone to embryonic lethality, and the resulting negative selections lead to evolutionary conservation of this phase. Here, we tested this “mid-embryonic lethality hypothesis” by measuring the rate of lethal phenotypes of three different species of vertebrate embryos subjected to two kinds of perturbations: transient perturbations and genetic mutations.

**Results:**

By subjecting zebrafish (*Danio rerio*), African clawed frog (*Xenopus laevis*), and chicken (*Gallus gallus*) embryos to transient perturbations, namely heat shock and inhibitor treatments during three developmental periods [early (represented by blastula and gastrula), pharyngula, and late], we found that the early stages showed the highest rate of lethal phenotypes in all three species. This result was corroborated by perturbation with genetic mutations. By tracking the survival rate of wild-type embryos and embryos with genetic mutations induced by UV irradiation in zebrafish and African clawed frogs, we found that the highest decrease in survival rate was at the early stages particularly around gastrulation in both these species.

**Conclusion:**

In opposition to the “mid-embryonic lethality hypothesis,” our results consistently showed that the stage with the highest lethality was not around the conserved pharyngula period, but rather around the early period in all the vertebrate species tested. These results suggest that negative selection by embryonic lethality could not explain hourglass-like conservation of animal embryos. This highlights the potential contribution of alternative mechanisms such as the diversifying effect of positive selections against earlier and later stages, and developmental constraints which lead to conservation of mid-embryonic stages.

**Electronic supplementary material:**

The online version of this article (10.1186/s13227-018-0095-0) contains supplementary material, which is available to authorized users.

## Background

Morphological novelty is realized through the alteration of developmental programs during evolution [1], and general tendency of the embryonic evolution has long been one of the major issues in the field of EvoDevo. As attempts to formulate the relationship between development and evolution, early studies predicted that the earliest developmental processes would tend to be conserved through evolution, because they establish fundamental information required for later developmental processes [2–4]. This idea was formulated independently in the “stepping stone model” [2], “developmental burden,” [3] and “generative entrenchment” [4]. In contrast, the highly similar morphological features of the mid-embryonic period in vertebrates have been recognized since the nineteenth century [5], and the “developmental hourglass model” [6, 7] predicts that the mid-embryonic phase is the most conserved stage in embryogenesis. It has also been predicted that anatomical patterns shared in this conserved mid-embryonic phase define a body plan for each animal phylum, and thus this phase has been named the phylotypic period [6–10]. Though no consensus has been reached as to which period is the most conserved in morphological perspective [11–14], recent transcriptomic studies support hourglass-like divergence patterns in embryogenesis in a variety of animal groups: The highest conservation has been reported in the mid-embryonic periods in vertebrates [15–22], arthropods (*Drosophila* species and *Anopheles gambiae*) [21, 23, 24], nematodes [25], and molluscs and annelids [26]. Furthermore, conserved molecular features of genes expressed in mid-developmental phases have also been reported in other multi-cellular organisms such as plants (*Arabidopsis thaliana*) [27] and fungi (*Coprinopsis cinerea*) [28]. However, the exact mechanisms that make embryos follow hourglass-like divergence remain to be clarified.

One possible evolutionary mechanism for hourglass-like conservation at transcriptomic level is that less-positive selections act on embryos during the mid-embryonic period than during earlier or later periods. For example, Slack et al. [29] referred to variability of earlier periods caused by adaptation to reproductive strategies or to the demands of embryonic nutrition. Similarly, Kalinka and Tomancak [9] proposed that diversifications of earlier and later periods can be attributed to adaptations to ecological niches. However, the absence of positive selections toward mid-embryonic period may not be sufficient to explain the hourglass-like divergence. For example, without negative selections, rapid neutral evolution [30, 31] could diversify the mid-embryonic period. Accordingly, Raff hypothesized that because the molecular signals in mid-embryos consist of complex inductive interactions among organ primordia, changes in the developmental processes during this period would likely cause lethal phenotypes, and thus there would be strong negative selection to conserve the mid-embryonic period [7]. Another possibility is that smaller phenotypic deviation is produced in the mid-embryonic period: A recent study has shown smaller transcriptomic deviation in the conserved mid-embryonic period of nematode strains that were established under the condition of near-absence of positive selections (realized by randomly selecting a single hermaphrodite at each generation to propagate) [32]. While the study implies that mid-embryonic conservation can be observed without positive selections, it does not preclude a possibility that negative selections contributed to the hourglass-like divergence of animal embryos. Given these, it is thus worth exploring other mechanisms, such as negative selection by embryonic lethality which might contribute to the transcriptomic conservation of the mid-embryonic period.

Here, we asked whether strong negative selection by embryonic lethality acts on the conserved mid-embryonic period of vertebrates (pharyngula period [18–21]). In pioneering research, Galis and Metz [33] paved the way to evaluating the contribution of negative selections by measuring prenatal death and abnormalities of embryos. By performing a meta-analysis of the susceptibility of various developmental stages to teratogens in rodent embryos, they reported that teratogen-induced death and abnormalities were highest during the pharyngula period, implying that strong negative selections act on this period [33]. However, embryonic death during early developmental periods could have been underestimated, since the papers assessed by this report used one of the eutherian group, namely rodents, and technical problems associated with estimation of prenatal death in eutherians have been identified [34]. To measure the embryonic lethality throughout embryogenesis, we utilized three non-eutherian vertebrate species in which we can reliably detect early death [zebrafish (*Danio rerio*), African clawed frog (*Xenopus laevis*), and chicken (*Gallus gallus*)]. We first measured their embryonic lethality following transient perturbations (i.e., inhibition of transcription, translation, and heat shock) and evaluated stage-specific lethality. Since genetic mutations are important sources of changes in embryogenesis in evolutionary processes, we also measured embryonic death rate under a condition expected to generate genetic mutations.

## Methods

### Animal care and embryo sampling

Riken wild-type (RW) zebrafish were maintained at 28.5 °C on a 14 h light: 10 h dark cycle. Embryos were obtained by natural mating and incubated at the same temperature as adults in 1/3 Ringer’s solution. The embryos were staged according to standard criteria outlined previously [35]. Female African clawed frogs were purchased from a local breeder (Watanabe Zoshoku, Hyogo, Japan), and males were purchased from another local breeder (Zenopasu Yoshoku Kyozai, Ibaraki, Japan). The eggs were artificially fertilized, dejellied in 2% w/v l-cysteine-HCl (pH 7.8), and reared in 0.1× Steinberg’s solution at 18 °C. Embryos were staged according to the standard table [36]. Fertilized chicken eggs were purchased from a local supplier (Shiroyama farm, Kanagawa, Japan) and incubated at 38 °C. The embryos were staged according to the criteria outlined previously [37]. Each replicate consisted of a population of embryos from different parents. All animal care and experimental procedures were performed in accordance with the protocols approved by the Animal Experiment Committee of the University of Tokyo (approval code: 14-3). All efforts were made to minimize suffering.

### Heat shock treatment

For heat shock treatment, zebrafish embryos were incubated at 39 °C for 5 h, African clawed frog embryos were incubated at 32 °C for 4 h, and chicken embryos were incubated at 45 °C for 4 h, respectively. The heating temperatures were the median lethal dose (LD_50_) value for each species. This dose was chosen to obtain sufficient statistical power to detect differences in rates of lethal phenotypes. To ensure that all cells of each embryo reach the same temperature, we sought the longest incubation time that could be applied equally to different developmental stages. Based on this criterion, we heated for the time length required for the gastrulation period, namely 5, 4, and 4 h for zebrafish, African clawed frog, and chicken embryos, respectively. The dead embryos of zebrafish and African clawed frog were removed daily and both before and after heat shock treatment to prevent deterioration of the incubation environment.

### Inhibitor treatment

Trichostatin A (TSA), an inhibitor of histone deacetylase I and II (Cayman Chemical, MI, USA); 17-allylamino-17-demethoxygeldanamycin (17-AAG), an inhibitor of Hsp90 (heat shock protein 90; Focus Biomolecules, PA, USA); and α-amanitin, an inhibitor of eukaryotic nuclear RNA polymerases II (Wako Pure Chemical Industries, Osaka, Japan) were used to transiently perturb the embryos. For zebrafish and African clawed frog embryos, each inhibitor was added to the incubation solution at various developmental points and the embryos were incubated for 5 and 4 h, respectively. The treatment length and inhibitor concentration were decided as described for the length and temperature of heat shock treatment; however, we did not measure rate of lethal phenotypes in African clawed frog embryos with α-amanitin treatment, because the specified concentration did not reach LD_50_.

Following inhibitor treatment, the embryos were washed three times in fresh incubation solution, placed into new dishes with fresh incubation solution, and reared until evaluation. Zebrafish embryos were dechorionated by 5 mg/ml pronase (Kaken Pharmaceutical Company, Tokyo, Japan) treatment at the 512-cell or 1 k-cell stage to promote inhibitor penetration. All chicken eggs were windowed and sealed with transparent packing tape 24 h after the start of incubation. A 500-µl volume of sterile Tyrode’s buffer containing 3% v/v penicillin and streptomycin (Pen Strep 10,000 U/ml; Gibco, Thermo Fisher Scientific, MA, USA) was applied to the embryos to avoid bacterial infection. Tyrode’s buffer containing TSA was applied to the embryos *in ovo* at various embryonic stages according to a previous study [38]. Doses used and treated stages are summarized in Tables 1 and 2, respectively. All control groups consisted of embryos incubated under standard conditions.

**Table 1:** Concentrations of inhibitors and temperatures used in heat shock experiments

	Heat shock (°C)	TSA	17-AAG (µM)	α-Amanitin
Zebrafish	39	500 nM	25	75 µg/ml
African clawed frog	32	1 µM	50	
Chicken	45	100 µM

**Table 2:** Developmental stages at which perturbations were initiated

	Earliest	←	→	Latest	Evaluation of rate of lethal phenotypes
Zebrafish	Sphere	30%-epiboly	24 hpf	48 hpf	hatch
African clawed frog	st. 8	st. 11	st. 28	st. 40	st. 45
Chicken	HH5		HH16	HH20	HH25

The pharyngula periods of zebrafish, African clawed frog, and chicken are 24 hpf (hours post-fertilization), st. 28 (stage 28), and HH16 (Hamburger Hamilton stage 16), respectively

### Evaluation of rate of lethal phenotypes

Rate of lethal phenotypes was evaluated at the hatch stage in zebrafish, at st. 45 in African clawed frog, and at HH25 in chicken. Rate of lethal phenotypes was defined as follows: (number of embryos with severe malformations in a survivor population + cumulative number of embryonic deaths)/(total number of embryos); severe malformation was included in the calculation because embryos with severe abnormality at birth are not expected to contribute to the next generation. Embryonic death was defined as follows: opaque appearance, collapse of tissue structure such as massive leak of cells from epidermis, a halt in the progression of embryogenesis, no heart beat or blood circulation, or no hatching. Malformation was defined as a halt in the progression development of one or more developmental organs (e.g., small eyes, abnormal head, short tail), edema, or bent body axis and was characterized according to criteria outlined in previous studies [39, 40]. In zebrafish and African clawed frog, movement defects were also included as abnormal phenotypes.

All data represent the mean value (+ SD) of two to five biological replicates consisting of at least 30 embryos. Statistical significances of differences in rate of lethal phenotypes were tested by multiple comparisons with the Tukey–Kramer method. An *α* level of 0.05 was used to define statistical significance.

### Immunohistochemistry

Immunohistochemistry was performed to compare the histone acetylation level between African clawed flog embryos treated with TSA at st. 40 and the control group. The embryos were fixed with MEMFA [0.1 M morpholinepropanesulfonic acid (MOPS), 2 mM ethylene glycol tetraacetic acid (EGTA), 1 mM MgSO_4_, and 4% formaldehyde], chopped with a razor, and dehydrated in an ethanol series. After replacement of ethanol with TBT buffer (50 mM Tris–Cl, 150 mM NaCl, 0.2% w/v bovine serum albumin, 0.1% v/v Triton X-100), the embryos were blocked with TBT buffer containing 2% bovine serum albumin (BSA) and then incubated with primary antibody (anti-histone H3 acetyl K27 antibody; Abcam, Cambridge, UK) overnight at 4 °C. After reaction with the secondary antibody, an ABC (avidin–biotin complex)–DAB (3,3′-diaminobenzidine) reaction was conducted for visualization.

### UV-C irradiation

Zebrafish and African clawed frog embryos were exposed to 254 nm UV (UV-C) light at 150 µJ/cm^2^. UV light of this wavelength induces DNA damage, such as pyrimidine dimers, which results in genetic mutations [41], and is widely used for mutagenesis experiments [41, 42]. UV intensity was measured with a UV meter (Solarmeter Model 8.0 UVC Meter, Solar Light Company, Inc., PA, USA). UV dose was adjusted by changing exposure time length, with constant intensity. To avoid overestimation of early embryonic mortality arising from mutations in maternal effect genes, stages around MZT (maternal-to-zygotic transition) initiation [43] were chosen for the UV irradiation. Zebrafish embryos at 512-cell stage and 24 hpf were exposed to UV-C for 4 s. This dose induced a significant increase in embryonic death rate (*P* = 1.14 × 10^−3^, log-rank test; and *P* = 1.05 × 10^−3^, Peto–Peto modified Gehan–Wilcoxon test), but induced a rate of embryonic death lower than the LD_50_ value. This dose was chosen to avoid difficulty in analysis in the late developmental period due to too few surviving embryos. For African clawed frog embryos, st. 9 was selected and the embryos were exposed to UV-C for 36 s. UV dose was decided for the same reason as for zebrafish, and UV irradiation significantly reduced the survival rate (*P *= 1.35 × 10^−3^, log-rank test; *P* = 4.86 × 10^−3^, Peto–Peto modified Gehan–Wilcoxon test).

### Survival rate tracking

Approximately 2 h after UV treatment, the zebrafish and African clawed frog embryos were transferred to 96-well plates with one embryo/well and incubated at 23 °C. Because zebrafish embryos at the 512-cell stage and African clawed frog embryos at st. 9 sometimes exhibit mass embryonic death for unknown reasons (even with no UV treatment), survival rate evaluation was started from Dome to 30%-epiboly in zebrafish, and st. 10 in African clawed frog (approx. 2 h after the UV-C treatment). The zebrafish embryos irradiated at 24 hpf were treated in the same way. Multi-position time-lapse imaging to track survival was performed at 1-h intervals by using a Leica AF 6000 fluorescence imaging system with a motorized stage and 5× air lens. Embryonic death was characterized by occurrence of severe deformations, such as cell mass extrusion and severe edema; malformations that become obvious only after hatching, such as curled body axis, were not included in embryonic death (Additional file 1: Figure S1). The total numbers of zebrafish embryos were 72 for the control group, 216 for the irradiated at 512-cell group, and 50 for the irradiated at 24 hpf group. In African clawed frog, the control group consisted of 72 embryos and the irradiated group consisted of 108 embryos. The difference in survival curves between treated and control embryos was tested by the log-rank test, which gives equal weight to all deaths at each time point, and by the Peto–Peto modified Gehan–Wilcoxon test, which gives more weight to deaths at early time points. Stage-specific death rate at any arbitrary developmental stage was calculated as the number of embryonic deaths occurring in the observed stage divided by the total number of survivors in the previous stage. The difference in the stage-specific death rates was tested by Fisher’s exact test with *P* value adjustment by the Holm method.

### Statistical tests

Biological replicates consisted of embryos from different parents to represent the general population of each developmental stage. For statistical tests, *α* level 0.05 was employed unless otherwise specified. To avoid an inflated type I error rate in multiple comparisons following ANOVA, we employed the Tukey–Kramer method for comparison of means and the Holm method *α*-level adjustment for comparison of ratios using Fisher’s exact test. To test the increase in embryonic lethality, both one-tailed testings were performed. Before the Tukey–Kramer method, Bartlett’s test (*α* = 0.01) was performed to verify the equal variances. Statistical analyses and plotting were performed using R (http://www.R-project.org/) [44], including the R packages survival [45, 46] and fmsb  [47] for survival analysis.

## Results

### Comparison of rates of lethal phenotypes after transient perturbations at different developmental stages

**Fig. 1:**
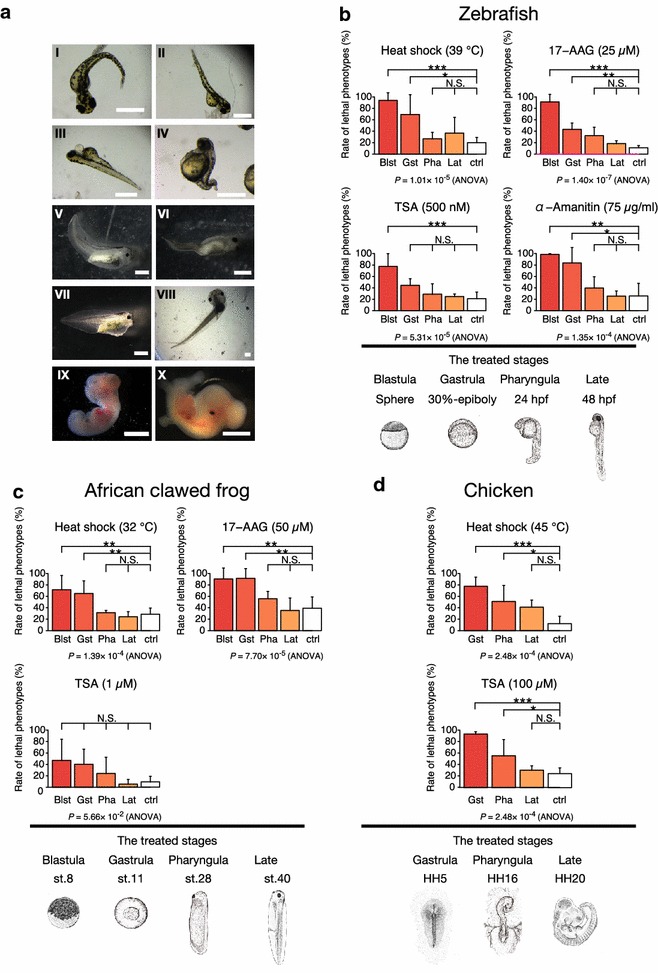
Pharyngula stages did not show the highest rate of lethal phenotypes following transient perturbations. **a** Representative embryos that showed malformation or lethal phenotype. (I–IV) Zebrafish embryos with (I) curled trunk, (II) bent trunk axis, (III) pericardial edema, and (IV) shortened trunk. (V–VIII) African clawed frog embryos with (V) curled trunk and small eyes, (VI) severely bent trunk, (VII) edema and abnormal head, and (VIII) bent trunk axis. (IX, X) Chicken embryos with (IX) abnormal head and growth arrest, and (X) small eyes. Scale bars represent 1 mm (I–VIII) and 5 mm (IX, X). **b**–**d** Rate of lethal phenotypes rate after transient perturbation in **b** zebrafish, **c** African clawed frog, and **d** chicken. Phenotype evaluation was performed at hatch period in zebrafish, st. 45 in African clawed frog, and HH25 in chicken. *Blst* blastula, *Gst* gastrula, *Pha* pharyngula, *Lat* late embryo, *ctrl* untreated control group. Data are displayed as means, and error bars denote SD. Only significant differences between each treated group and the control group are shown. *P < 0.05, **P < 0.01, ***P < 0.001 (Tukey–Kramer method)

To identify the developmental period most susceptible to changes in developmental process caused by transient perturbations, we subjected embryos to transient treatment at different developmental stages and compared rate of lethal phenotypes (Fig. 1). In evaluating the rate of lethal phenotypes, we included not only dead embryos but also embryos with severe abnormality (Fig. 1a), as these embryos are not expected to contribute to the next generation. To avoid bias, we selected treatments that are expected to induce global disturbances at all developmental stages rather than a specific stage. Namely, we used heat shock and the following inhibitors for the transcriptional and translational processes: TSA, an inhibitor of histone deacetylases, which have crucial roles in transcriptional control [48]; 17-AAG, a potent inhibitor of Hsp90, an essential protein for protein folding and transcription regulation [49–52]; and α-amanitin, a transcriptional inhibitor targeting RNA polymerase II [53]. Efficient penetration of TSA into the center of the embryonic body was confirmed by immunostaining of acetylated histone in the late African clawed frog embryo (Additional file 2: Figure S2). We also confirmed that the 17AAG-induced abnormalities resembled those reported in a previous study of 17-AAG-treated gastrulas [54], and making windows in the shell of chicken egg did not affect lethality of chicken embryos (Additional file 3: Figure S3).

If the “mid-embryonic lethality hypothesis” is correct, embryos treated in the pharyngula period should show the highest rates of lethal phenotypes. However, zebrafish embryos treated in the blastula period (around Sphere) showed significantly higher rates of lethal phenotypes than the control (untreated) group in all three transient perturbations tested (Fig. 1b; heat shock, *P* = 1.24 × 10^−4^; TSA, *P* = 3.72 × 10^−4^; 17-AAG*, P* = 0.5 × 10^−7^; α-amanitin, *P* = 2.30 × 10^−3^; Tukey–Kramer method). In contrast, embryos treated in the pharyngula period (around 24 hpf) did not show a significantly higher rate of lethal phenotypes than the control group (*P* > 0.05, Tukey–Kramer method). Similarly, embryos of the African clawed frog subjected to heat shock or 17-AAG treatment in the pharyngula period (around st. 28) did not show a significantly higher rate of lethal phenotypes than the control embryos (*P* > 0.05, Tukey–Kramer method), whereas those treated during the blastula (around st. 8) or gastrula (around st. 11) period showed a significantly higher rate of lethal phenotypes than the control embryos (Fig. 1c; heat shock, *P* = 4.67 × 10^−3^; 17-AAG, *P* = 4.95 × 10^−3^, Tukey–Kramer method). Embryos treated with TSA showed a similar trend, but it was not statistically significant (*P* = 0.057, ANOVA). To further confirm the low lethal tendency of the pharyngula period, we have performed analysis of the rate of lethal phenotypes in chicken embryos. In chicken embryos, rate of lethal phenotypes was significantly higher than the control group in both the embryos treated in the gastrula period (around HH5) (Fig. 1d; heat shock, *P* = 4.63 × 10^−4^; TSA, *P* = 1.27 × 10^−4^, Tukey–Kramer method) and those treated in the pharyngula period (around HH16) (heat shock, *P* = 1.19 × 10^−2^; TSA, *P* = 1.02 × 10^−2^, Tukey–Kramer method); however, as in the other species, the rate of lethal phenotypes was not different between the pharyngula period and the gastrula period (*P* > 0.05, Tukey–Kramer method). Note that the blastula period was not tested in chicken.

### Tracking survival rates following UV irradiation

**Fig. 2:**
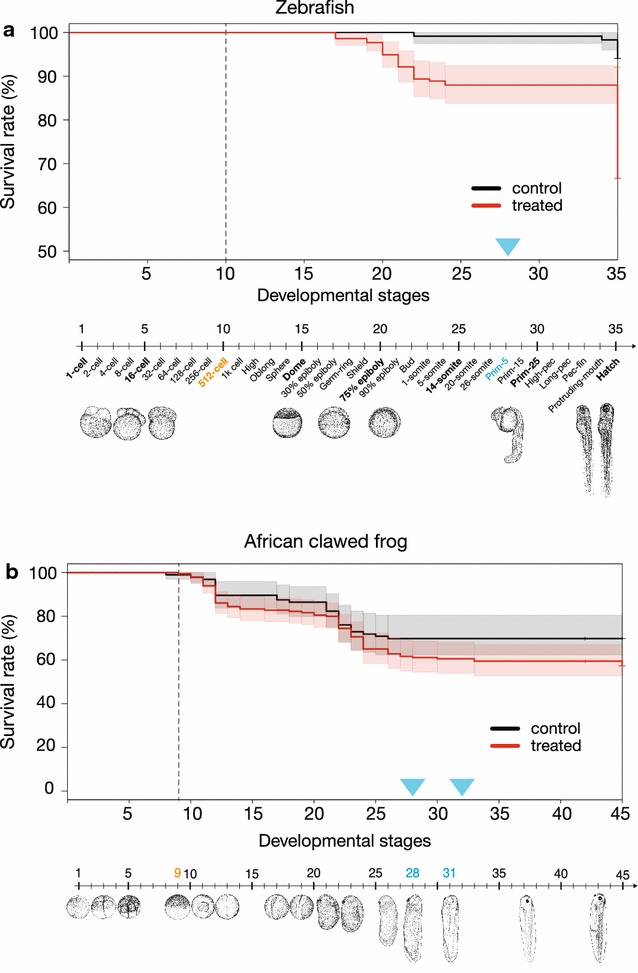
Survival rate after UV irradiation decreased in the gastrula period, but not the pharyngula period. Kaplan–Meier survival curves of **a** zebrafish and **b** African clawed frog embryos after UV irradiation at the MZT period. Note that the horizontal axis shows successive developmental stages rather than actual time length. For zebrafish, developmental stages described by Kimmel et al. [35] were numbered sequentially from 1 to 35. Images of the developmental stages are shown below, with the numbers on the line corresponding to those in the survival curve *x* axis. Blue arrowheads, most conserved developmental periods in previous reports [18, 20]; black line, control; red line, UV-irradiated embryos; shaded area, 95% CI (confidence interval); vertical dotted line, UV irradiation. Numbers of embryos used in this analysis: zebrafish control group, *n* = 72; zebrafish treated group, *n* = 216; African clawed frog control group, *n* = 72; and African clawed frog treated group, *n* = 108

Negative selection against embryos does not result only from transient perturbations, but also results from genetic mutations. Since genetic mutations are the primary source of heritable changes in embryogenesis through evolution, we next tested whether the pharyngula embryos have high susceptibility to genetic mutations. To this end, we exposed embryos to UV-C (254 nm, the wavelength widely used for mutation induction [41, 42]) around the maternal-to-zygotic transition (MZT) and measured their survival rate throughout embryogenesis (Fig. 2a, b; Additional file 1: Figure S1).

In zebrafish, if the “mid-embryonic lethality hypothesis” is correct, the highest embryonic death rate should be observed around the conserved developmental period (24 hpf [18, 20]), which is marked as stage 28 [Prim-5] in Fig. 2a, horizontal axis. However, reduced survival rates in the UV-irradiated group were detected only around the gastrula and early segmentation periods (from 50%-epiboly to 5-somite stage); no embryonic death was observed in the pharyngula period (from Prim-5 to High-pec). In addition, we confirmed that the increase in embryonic death rate was not due to the temporal proximity to UV irradiation, because the survival curve of zebrafish embryos exposed to UV in the pharyngula period did not differ significantly from that of the control embryos (*P* = 0.136, log-rank test; *P* = 0.137, Peto–Peto modified Gehan–Wilcoxon test) (Additional file 4: Figure S4). A similar trend for embryos exposed at the MZT was observed in the experiments using African clawed frog embryos (Fig. 2b). While embryonic death was observed at almost all stages (no incidence at st. 17 or st. 25), a higher embryonic death rate was observed at stages much earlier (e.g., st. 12, late gastrula period) than the previously identified conserved periods (st. 28 and st. 31 [18, 20]). By analyzing stage-specific embryonic death rate, both conserved periods (st. 28, 1/111 [0.9%] and st. 31, 0/109 [0%]) showed significantly lower embryonic death rate than that at st. 12 (st. 12, 14/169 [8.3%]; st. 28 vs st. 12, *P* = 6.0 × 10^−3^; st. 31 vs st. 12, *P* = 1.7 × 10^−3^; Fisher’s exact test adjusted by Holm method). Note that the embryonic death rate of earlier stages (st. 12) was also higher than the conserved stages in control group of African clawed frogs (st. 12, 7/93 [7.5%]; st. 28 and st. 31, 0/68 [0%]; *P* = 2.9 × 10^−2^; Fisher’s exact test). However, the tendency was not corroborated in zebrafish embryos (Bud, 1/118 [0.8%]; Prim-5, 0/117 [0%]; *P* = 1.0; Fisher’s exact test). In summary, the period of high embryonic death was observed around the gastrula period in zebrafish, and the gastrula and early tailbud periods in African clawed frog. Contrary to the “mid-embryonic lethality hypothesis,” the highest reduction in survival rate was not observed during the pharyngula period in either species.

## Discussion

Recent transcriptomic studies have revealed that the mid-embryonic stages, especially around pharyngula stages, are highly conserved in vertebrates [18–22], supporting the developmental hourglass model [6, 7]. Although the key mechanisms of this hourglass-like conservation remain unclarified, one attractive hypothesis attributes hourglass-like conservation to high rates of lethality induced in the pharyngula period (“mid-embryonic lethality hypothesis”) [7, 33]. Here, we tested this hypothesis by measuring the rate of lethal phenotypes at various embryonic stages following transient perturbations and genetic mutations.

We first treated embryos with inhibitors of key cellular proteins (histone deacetylase I and II, Hsp90, and eukaryotic nuclear RNA polymerases II) or heat shock to reach around LD_50_ as transient perturbations and measured rate of lethal phenotypes. The result showed that the rate of lethal phenotypes was higher in early embryos around the gastrula period than in pharyngula embryos in all perturbation conditions (Fig. 1). This was consistent in all three species tested, namely zebrafish, African clawed frog, and chicken (Fig. 1b–d). In contrast to the results expected, if the “mid-embryonic lethality hypothesis” were true, these findings indicate that vertebrate pharyngula period is not the phase with highest lethality. Of note, the control group in African clawed frog also showed a slight increase in embryonic death at stages earlier than the conserved stages (Fig. 2b). Our results, however, are not consistent with the conclusion reached by a previous report [33] based on meta-analysis of rodents. That report showed that the developmental stages that are most susceptible to lethal effects of teratogens in rodents are the pharyngula stages. While the exact reason for this discrepancy remains to be clarified, there are several possible explanations. One is the potential underestimation of early embryonic death in rodents [34] in the original papers. The differences in chemical agents and species analyzed may also explain the inconsistency of the lethal tendencies. A caveat in our approach is that the perturbation sources we utilized may not reflect those under natural conditions. For example, the high rate of lethal phenotypes in the earlier periods induced by the inhibitors could in part be due to cell cycle arrest [55]. However, considering the consistency in our results across perturbation sources and species, it seems likely that the earlier phase is also susceptible to perturbations under natural conditions. Another caveat is that the treatments with LD_50_ doses may be a much exaggerated perturbation compared with those under natural conditions. Meanwhile, higher embryonic death rates in the early developmental period compared to conserved stages were also observed in the untreated African clawed frog embryos (Fig. 2b), and perturbations under natural conditions may possibly also induce high lethality in early developmental period. As the results were not corroborated in the zebrafish, further studies are required to test this tendency. The same tendency has been implied in humans: The greatest pregnancy loss appears to occur during the very early period of embryogenesis, namely around preimplantation and the first week of implantation [56], and teratogen exposure causes embryonic death during early development (i.e., the first weeks in embryogenesis), whereas it causes malformations during the mid-embryonic period [57].

Consistent with the transient perturbation experiments, a higher embryonic death rate was observed in the early period than in the pharyngula period in the experiments where embryos were irradiated at the MZT: The gastrula period in zebrafish and the gastrula and early tailbud periods in African clawed frog showed notable decreases in survival rate (Fig. 2). Importantly, UV exposure in the pharyngula period had no significant effect on survival (Additional file 4: Figure S4), suggesting that the high early embryonic death was not merely a result of the immediate effect of UV exposure, but rather was the consequence of UV-induced genetic mutations. These results suggest that early vertebrate embryos such as gastrula and early tailbud embryos are prone to show lethal phenotypes than pharyngula embryos when genetic mutations were induced. The main limitation of this approach is that irradiation was done at the multi-cell stage (see “Methods”) and therefore probably resulted in somatic mosaic mutations, which may not perfectly reflect mutations that occur under natural conditions. Analyzing a variety of transgenic lines would help to compensate for these limitations in our study. From this perspective, it is worth noting that a previous study characterized the phenotypes of 1751 knockout mouse lines [58], and showed that the embryonic death rate was higher in the earlier period than in the most conserved period in mice (E9.5, shown in [18, 20]). Our results are also consistent with those of a transcriptome-based study, which showed enrichment of expression of essential genes at early zebrafish developmental stages [59].

The observed patterns of lethality suggest that the evolutionary conservation of the pharyngula period cannot be explained solely by negative selections from mid-embryonic lethality, and further indicate the existence of as yet unknown mechanisms driving the mid-embryonic conservation in vertebrates. It has to be noted that our study is based on only three vertebrate species and that it is unclear whether the same tendency can be observed in other groups of animals that show hourglass-like conservation [21, 23–25]. Additionally, our study does not preclude potential contributions from lethal phenotypes after embryogenesis since we focused on extreme negative selections during embryogenesis and did not evaluate the overall fitness of the surviving embryos. In other words, mutations or perturbations that cause lethal or less adaptive phenotypes in the period after embryogenesis may have been overlooked. For example, Young et al. suggest that conservation of facial morphology among avian mid-embryos is not due to embryonic lethality but rather to selection against maladapted phenotypes, such as cleft lip [60]. As mentioned above, another attractive candidate for the mechanism behind the hourglass-like divergence is positive selection acting on embryos in early and late periods and promoting diversification [9, 29]. Developmental constraints could also be contributing to conservation of the mid-embryonic phase, although the mechanism remains to be clarified. In a recent study, Zalts and Yanai [32] observed smaller variation in gene expression during the mid-embryonic phase than during earlier and later phases in nematodes under near-absence of positive selection and proposed this as evidence of developmental constraint. While the exact mechanism that led to smaller transcriptomic variations during the mid-embryonic period of nematodes remains to be clarified, pleiotropic constraints [22, 61] could explain this limited transcriptomic variation. Finally, it is possible that certain molecular components that play important role in mid-embryonic period such as genes involved in cell–cell communication and homeodomain transcription factors [18, 32] or Tet (ten–eleven translocation) protein function [62] could also be contributing to mid-embryonic conservation. To date, the results are not conclusive, and further studies are needed to explore the exact mechanism that has led to the hourglass-like conservation observed in various animal lineages. In this regard, finding animal groups that do not follow the hourglass-like divergence would be important.

## Conclusion

The present study showed that vertebrate pharyngula embryos are less susceptible to transient perturbation and genetic mutations than are earlier embryos. The results suggest that negative selection from embryonic lethality could not explain the hourglass-like conservation observed among vertebrate embryos. The need to explore alternative mechanisms is highlighted.

## Additional files

**Additional file 1: Figure S1.** Developmental dynamics of zebrafish embryos showing normal development and examples of embryonic death. After UV irradiation, embryonic survival was tracked by time-lapse imaging in 1-h intervals until the hatch period (at least 60 h). The elapsed time from the start of recording is indicated in the upper left corner in each panel. **(a)** Entire time-lapse sequences of the typical normal development of a zebrafish embryo. **(b, c)** Examples of embryonic death; in these cases, we determined that embryonic death occurred at 6 h (**b**) and 19 h (**c**), respectively. In both frames, critical deformation and cessation of development were observed.

**Additional file 2: Figure S2.** Efficient penetration of TSA in African clawed frog late embryos. Immunohistochemistry was performed with the anti-histone H3 acetyl K27 antibody. **(a-1)**–**(f-1)** Lateral views of whole st. 40 embryos. Arrowheads indicate the position of the cross section and the observed plane. **(a-2)**–**(f-2)** Transverse section through the trunk of st. 40 embryos. Areas included in the magenta and green squares are shown at higher magnification in (**a-3**)–(**f-3**) and (**a-4**)–(**f-4**), respectively. nt, neural tube; nc, notochord. **(a-3)**–**(f-3)** Neural tube and notochord of st. 40 embryos. **(a-4)**–**(f-4)** Trunk of st. 40 embryos. The experiment was performed two times (*n* = 5 in each replicate). The results of each experiment are shown separately, as DAB staining reaction proceeds rapidly and staining intensity is variable among experiments.

**Additional file 3: Figure S3.** Windowing did not affect lethality in chicken embryos. Rate of lethal phenotypes of chicken embryos treated with TSA. Eggs were windowed and embryos were treated during the gastrula (Gst), pharyngula (Pha), and late (Lat) periods, or eggs were windowed but embryos not treated (Wnd). These are the same data shown in Fig. 1d. Additional data is the lethality of embryos that were not windowed or treated (Intct). Data are means; error bars are SD. *P < 0.05, **P < 0.01, ***P < 0.001 vs Gst; ^†^*P* < 0.05, ^††^*P* < 0.01, ^†††^*P* < 0.001 vs Pha (Tukey–Kramer method). Lethality in Wnd (24.5%) was not significantly different from that of Intct (8.8%; *P* = 0.09, Tukey–Kramer method), suggesting that the effect of windowing on lethality could be ignored in this experiment.

**Additional file: Figure S4.** Survival rate did not decrease after UV irradiation in the pharyngula period. Survival curves of zebrafish embryos after UV irradiation in the pharyngula period (Kaplan–Meier method). The horizontal axis is developmental stage and does not reflect actual time length. Orange arrowhead, most conserved developmental period in vertebrates [18, 20]. Black line, control (same group depicted in Fig. 2a); orange line, embryos UV irradiated in the pharyngula period (24 hpf = Prim-5); shaded area, 95% CI; vertical dotted line, UV irradiation. Numbers of embryos used in this analysis: control group, *n* = 72; treated group *n* = 50.

## Abbreviations

ABC: avidin–biotin complex
CI: confidence interval
DAB: 3,3′-diaminobenzidine
EGTA: ethylene glycol tetraacetic acid
HH: Hamburger Hamilton
hpf: hours post-fertilization
Hsp90: heat shock protein 90
LD_50_: median lethal dose
MOPS: morpholinepropanesulfonic acid
MZT: maternal-to-zygotic transition
st.: stage
Tet: ten–eleven translocation
TSA: trichostatin A
17-AAG: 17-allylamino-17-demethoxygeldanamycin
