# Supplementary figures and images for: Embryonic lethality is not sufficient to explain hourglass-like conservation of vertebrate embryos
Source: EvoDevo. 2018 Mar 16;9:7. doi: 10.1186/s13227-018-0095-0 (PMC5855935; doi:10.1186/s13227-018-0095-0)

**a**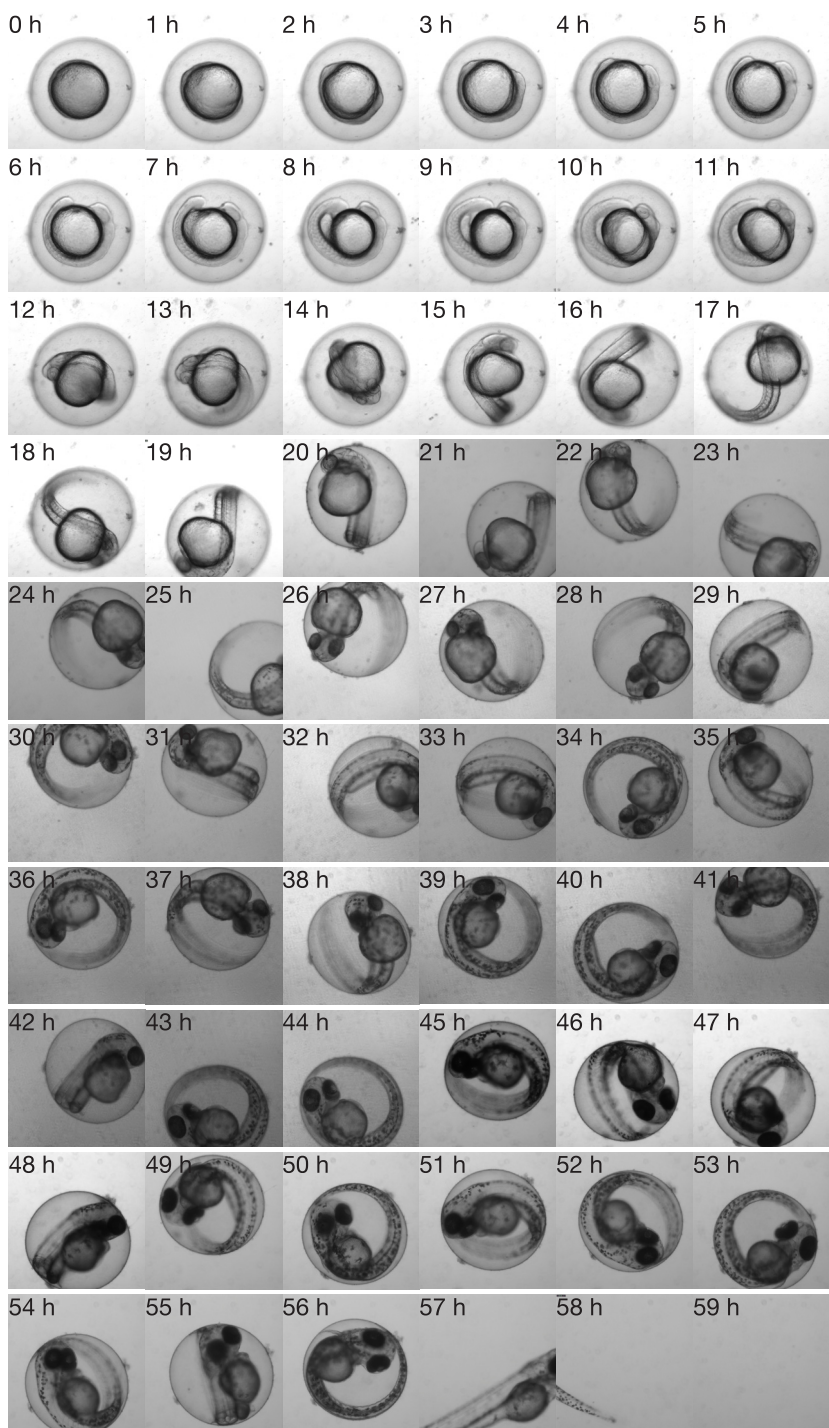**b**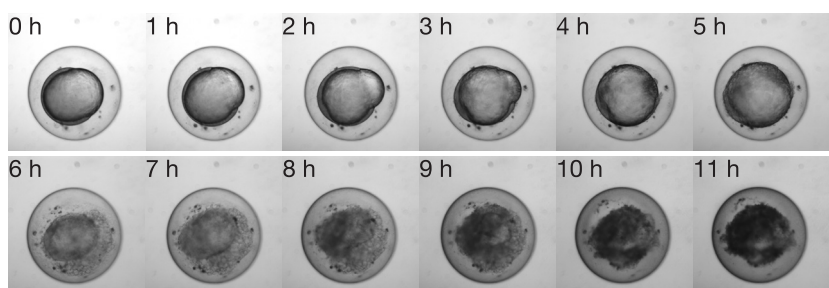**c**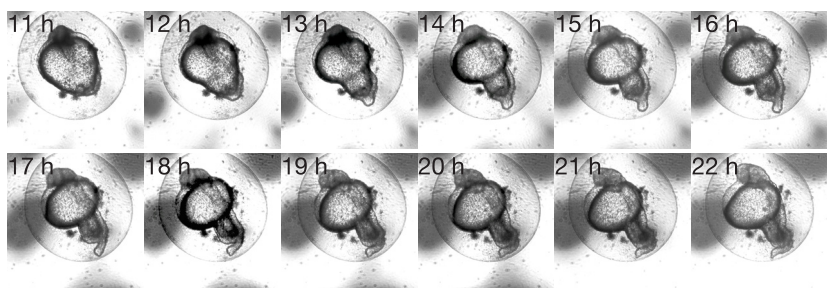

Supplement: Supplementary file 1 — Additional file 1: Figure S1. Developmental dynamics of zebrafish embryos showing normal development and examples of embryonic death. After UV irradiation, embryonic survival was tracked by time-lapse imaging in 1-h intervals until the hatch period (at least 60 h). The elapsed time from the start of recording is indicated in the upper left corner in each panel. (a) Entire time-lapse sequences of the typical normal development of a zebrafish embryo. (b, c) Examples of embryonic death; in these cases, we determined that embryonic death occurred at 6 h (b) and 19 h (c), respectively. In both frames, critical deformation and cessation of development were observed. [file 13227_2018_95_MOESM1_ESM.pdf]

replicate #1

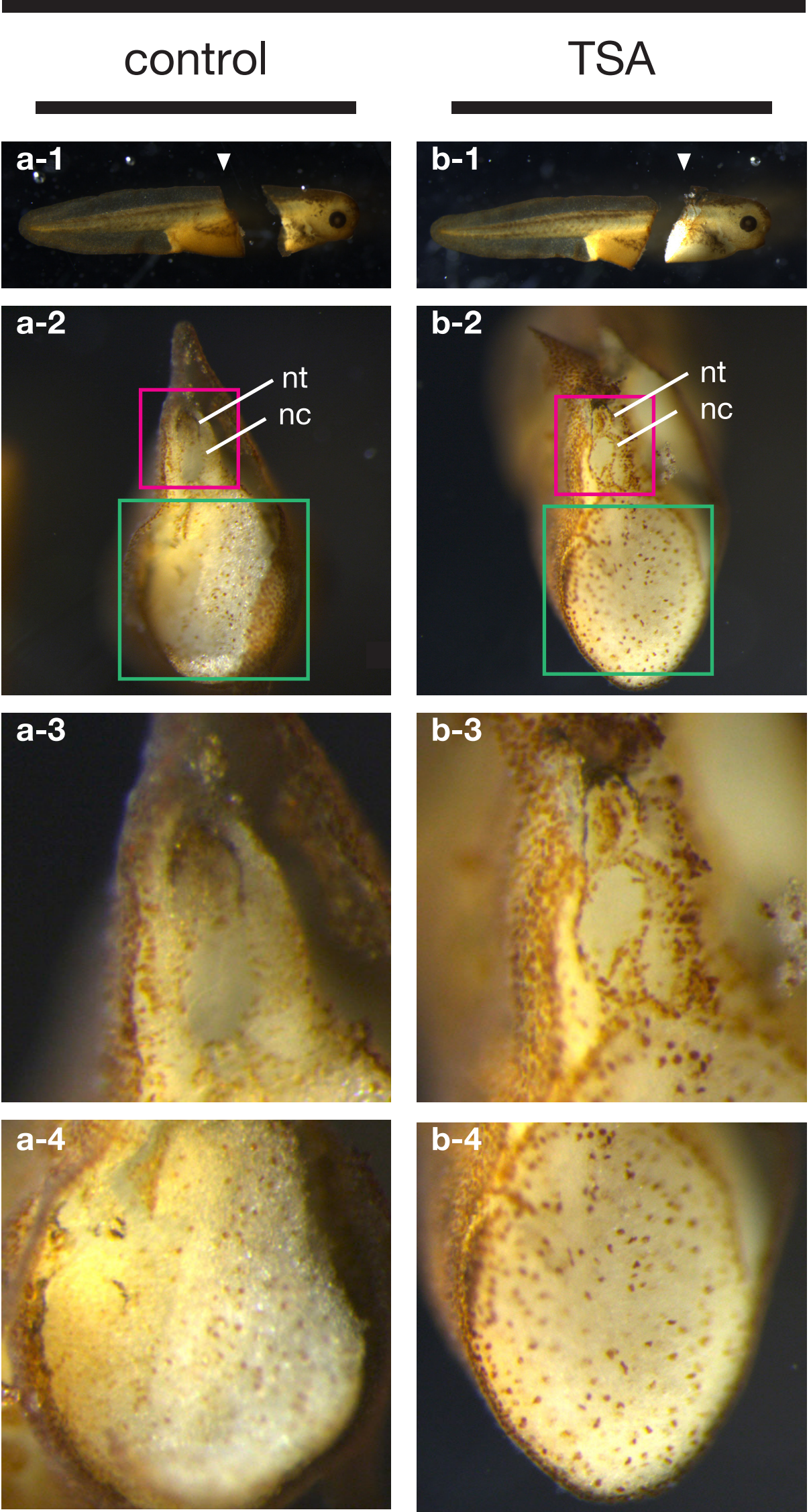

replicate #2

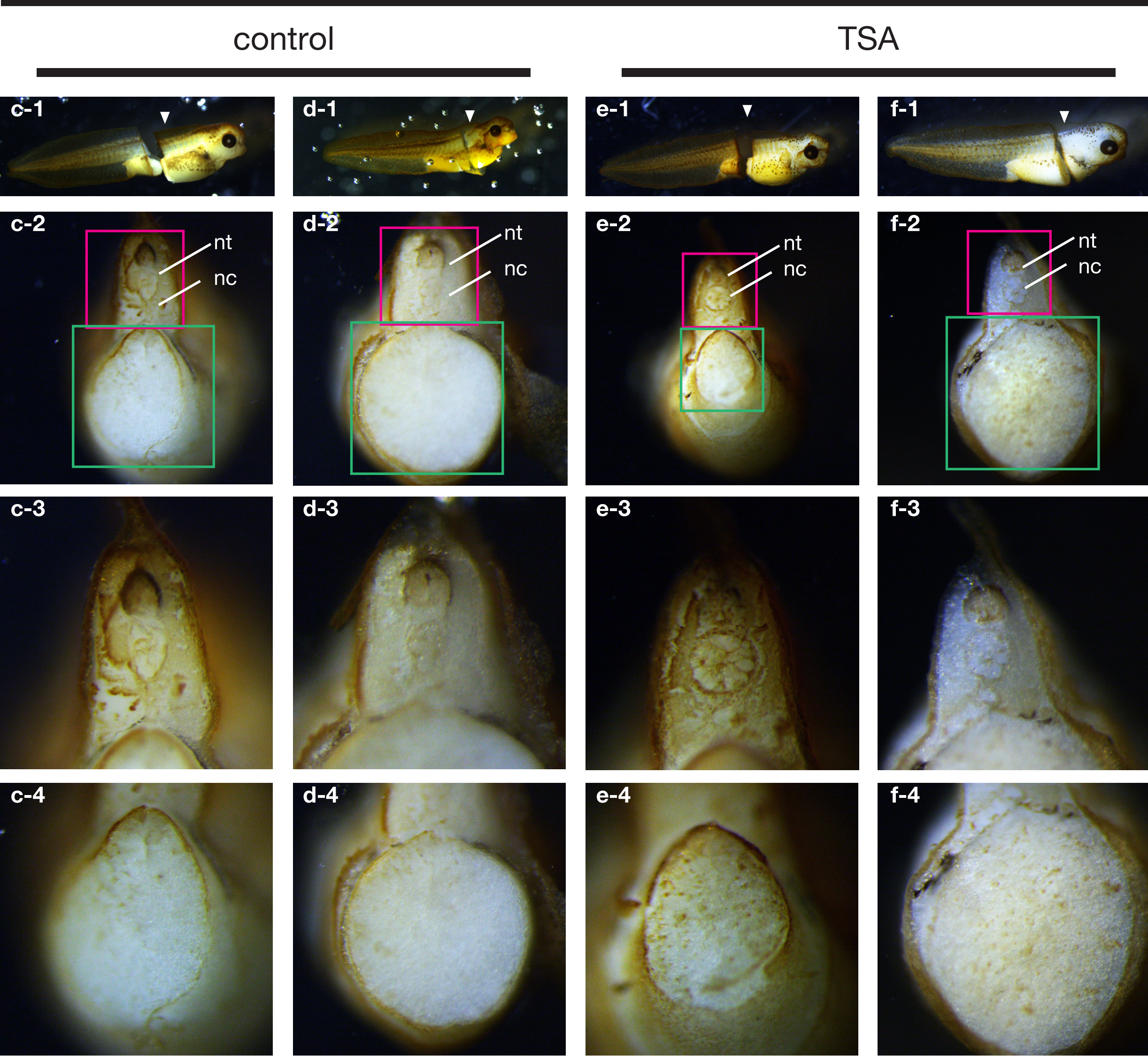

Supplement: Supplementary file 2 — Additional file 2: Figure S2. Efficient penetration of TSA in African clawed frog late embryos. Immunohistochemistry was performed with the anti-histone H3 acetyl K27 antibody. (a-1)–(f-1) Lateral views of whole st. 40 embryos. Arrowheads indicate the position of the cross section and the observed plane. (a-2)–(f-2) Transverse section through the trunk of st. 40 embryos. Areas included in the magenta and green squares are shown at higher magnification in (a-3)–(f-3) and (a-4)–(f-4), respectively. nt, neural tube; nc, notochord. (a-3)–(f-3) Neural tube and notochord of st. 40 embryos. (a-4)–(f-4) Trunk of st. 40 embryos. The experiment was performed two times (n = 5 in each replicate). The results of each experiment are shown separately, as DAB staining reaction proceeds rapidly and staining intensity is variable among experiments. [file 13227_2018_95_MOESM2_ESM.pdf]

# Zebrafish

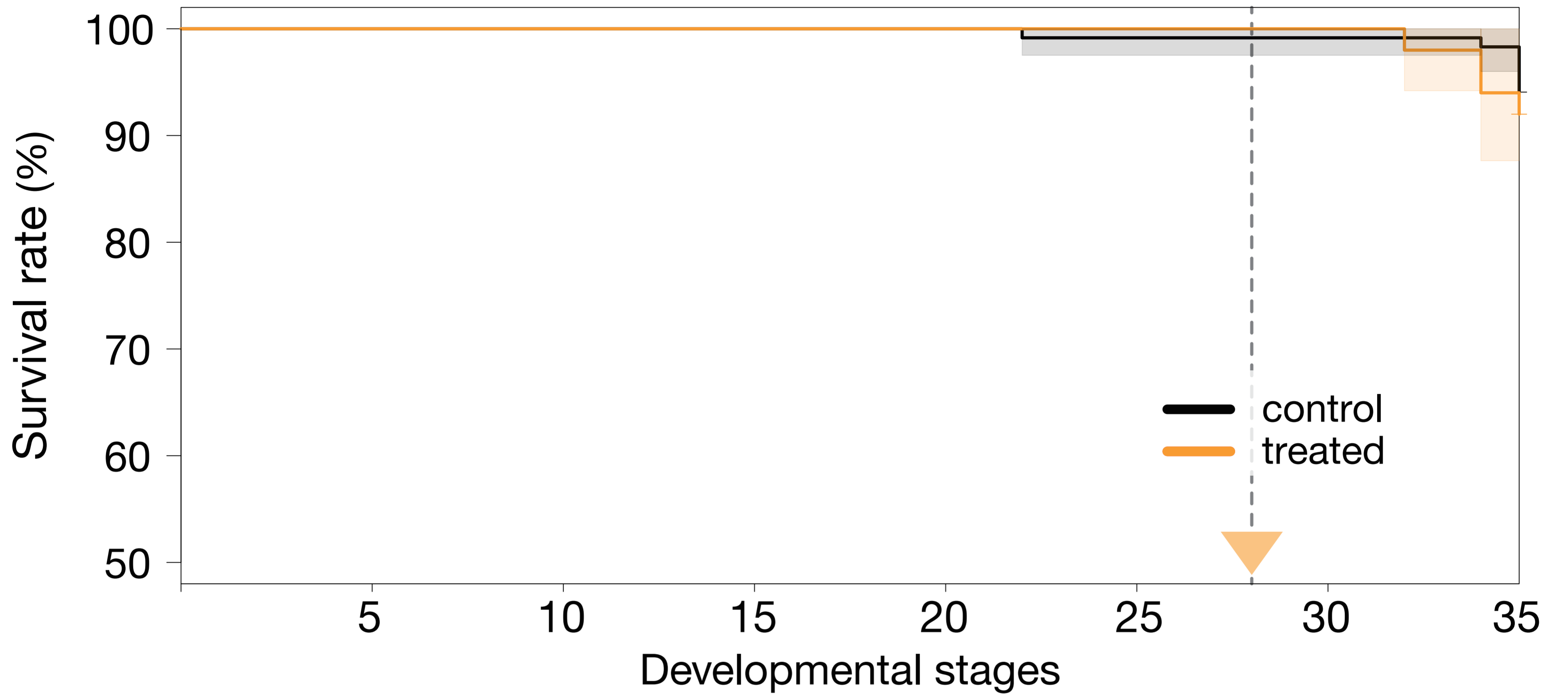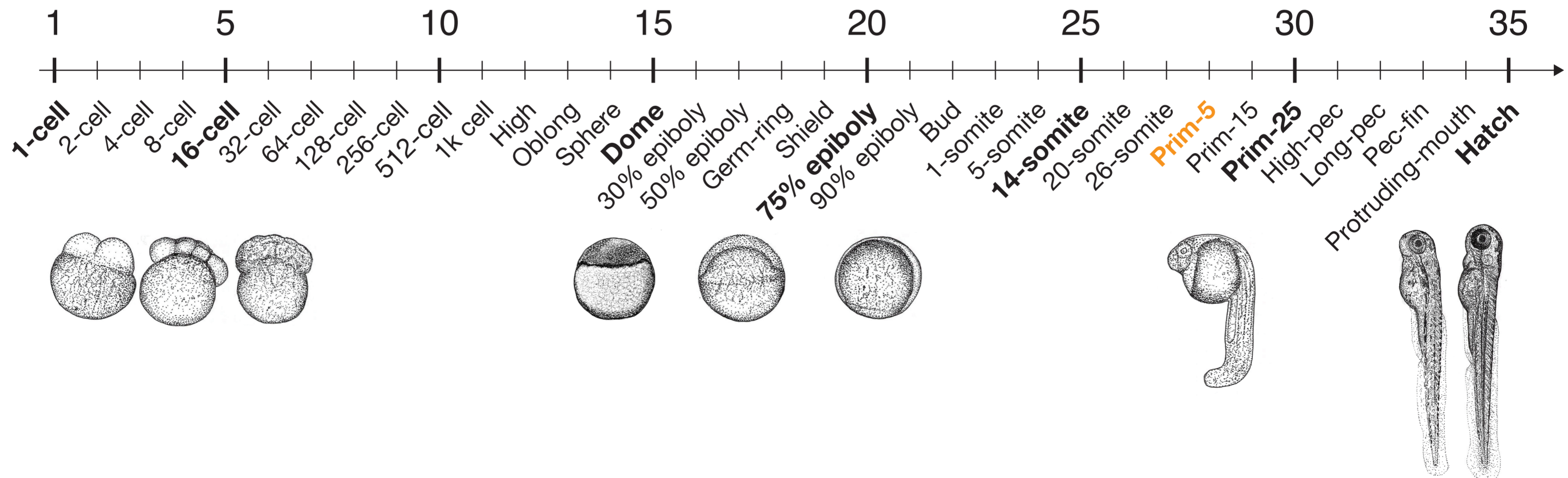

Supplement: Supplementary file 4 — Additional file: Figure S4. Survival rate did not decrease after UV irradiation in the pharyngula period. Survival curves of zebrafish embryos after UV irradiation in the pharyngula period (Kaplan–Meier method). The horizontal axis is developmental stage and does not reflect actual time length. Orange arrowhead, most conserved developmental period in vertebrates [18, 20]. Black line, control (same group depicted in Fig. 2a); orange line, embryos UV irradiated in the pharyngula period (24 hpf = Prim-5); shaded area, 95% CI; vertical dotted line, UV irradiation. Numbers of embryos used in this analysis: control group, n = 72; treated group n = 50. [file 13227_2018_95_MOESM4_ESM.pdf]
