# Supplementary material for: Embryonic lethality is not sufficient to explain hourglass-like conservation of vertebrate embryos
Source: EvoDevo. 2018 Mar 16;9:7. doi: 10.1186/s13227-018-0095-0 (PMC5855935; doi:10.1186/s13227-018-0095-0)
Supplement: Supplementary file 3 — Additional file 3: Figure S3. Windowing did not affect lethality in chicken embryos. Rate of lethal phenotypes of chicken embryos treated with TSA. Eggs were windowed and embryos were treated during the gastrula (Gst), pharyngula (Pha), and late (Lat) periods, or eggs were windowed but embryos not treated (Wnd). These are the same data shown in Fig. 1d. Additional data is the lethality of embryos that were not windowed or treated (Intct). Data are means; error bars are SD. *P < 0.05, **P < 0.01, ***P < 0.001 vs Gst; †P < 0.05, ††P < 0.01, †††P < 0.001 vs Pha (Tukey–Kramer method). Lethality in Wnd (24.5%) was not significantly different from that of Intct (8.8%; P = 0.09, Tukey–Kramer method), suggesting that the effect of windowing on lethality could be ignored in this experiment. [file 13227_2018_95_MOESM3_ESM.pdf]

Chicken

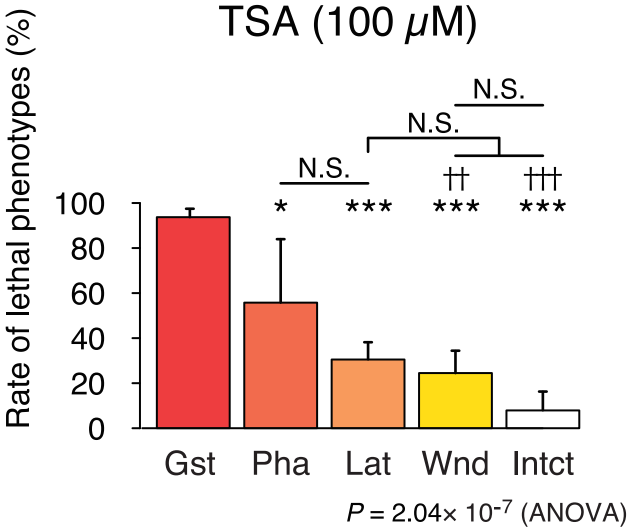

TSA (100 μM)
Rate of lethal phenotypes (%)
100
80
60
40
20
0
N.S.
N.S.
N.S.
*
***
††
***
†††
***
Gst
Pha
Lat
Wnd
Intct
P = 2.04× 10-7 (ANOVA)

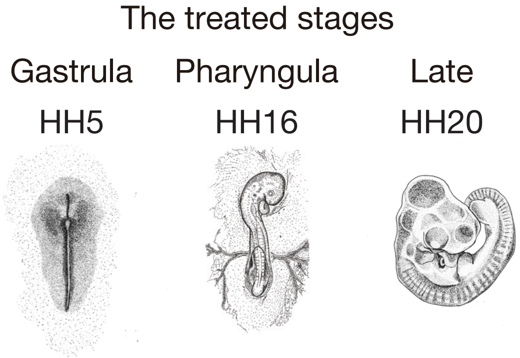

The treated stages
Gastrula
HH5
Pharyngula
HH16
Late
HH20
